# An Examination of Morphometric Variations in a Neotropical Toad Population (*Proceratophrys cristiceps*, Amphibia, Anura, Cycloramphidae)

**DOI:** 10.1371/journal.pone.0003934

**Published:** 2008-12-17

**Authors:** Kleber S. Vieira, Cristina Arzabe, Malva I. M. Hernández, Washington L. S. Vieira

**Affiliations:** 1 Departamento de Sistemática e Ecologia, Universidade Federal da Paraíba Campus I. João Pessoa, Paraíba, Brazil; 2 Embrapa Meio-Norte/UEP-Parnaíba, Parnaíba, Piauí, Brazil; 3 Departamento de Ecologia e Zoologia, Universidade Federal de Santa Catarina, Florianópolis, Santa Catarina, Brazil; 4 Programa de Pós-graduação em Ciências Biológicas (Área de Concentração em Zoologia) e Coleção Herpetológica do Departamento de Sistemática e Ecologia, Universidade Federal da Paraíba, Campus I, João Pessoa, Paraíba, Brazil; University of Bristol, United Kingdom

## Abstract

The species *Proceratophrys cristiceps* belongs to the genus *Proceratophrys* within the family Cycloramphidae. These amphibians are found exclusively in South America in the morphoclimatic domain of the semi-arid depression zones in northeastern Brazil known as the *Caatinga*. We examined intrapopulational variation using univariate and multivariate statistics with traditional and geometric morphometrics, which supported the existence of two morphotypes of this species. Our results indicated significant degrees of variation in skeletal characteristics between some natural populations of this species. Careful analyses of variability levels are fundamental to avoid taxonomic errors, principally in populations that demonstrate characteristics intimately associated with their area of occurrence, as is the case of *Proceratophrys cristiceps*.

## Introduction

Individuals of the species *Proceratophrys cristiceps* (Müller, 1883) are found exclusively in the inter-montane depression zones and inter-plain semi-arid regions known collectively as the *caatinga* (dryland) region [1]. These amphibians are classified within the family Cycloramphidae [2] and belong to the genus *Proceratophrys* Miranda-Ribeiro, 1920, together with 18 other species distributed within different exclusively South American morpho-climatic domains.

The species *P. cristiceps* was originally described based on external morphological characteristics as well as some skeletal features [3]. Information concerning this species is still relatively scarce overall, and the most important information beyond that presented in the original description can be found in only three other published works that examine their morphology [4], [2], [5]; information regarding their ecology and biology is essentially non-existent.

Studies that deal with chromatism (whether in amphibians or other taxa) generally describe such variations as being characteristics of distinct species or treat it as a question related to taxonomic identification [6], [7]-while other authors have utilized this character to distinguish populations according to the areas in which it occurs [8]. Two basic color patterns can be observed in *P. cristiceps* that always appear sympatrically throughout their range in the caatinga, a characteristic common to some other species of the same genus.

There are currently no published reports of other visual characteristics that would allow the identification of the morphotypes of *P. cristiceps* beyond their general color patterns. The present work examines intraspecific variation within a population of neotropical toads and identifies two morphotypes of the species *P. cristiceps* through the use of morphometric studies and the morphology of their crania, and discusses polymorphism and its implication in the taxonomy of these amphibians as well as other taxa.

## Results

Individuals of both morphotypes of *P. cristiceps* demonstrated significant differences in terms of eye-nostril distance (END) by the Student *t* test, with this variable character being on the average 0.26 mm larger than the common morphotype. No other statistically significant differences were observed in relation to the other measurements between the two morphotypes (Table 1).

**Table 1:** Student *t* test of the morphotype measurements of *P. cristiceps*.

Variables	Common morphotype		Yellow morphotype		t	DF	p	Variances	p Levene
	Average±SD	N	Average±SD	N					
LogSVL	3.20±0.503	59	3.11±0.415	20	0.78	39	0.44	1.47	0.47
LogHW	2.42±0.465	59	2.43±0.503	20	−0.01	31	0.99	1.17	0.62
LogIND	0.64±0.410	59	0.51±0.324	20	1.37	41	0.18	1.60	0.13
**LogEND**	**0.77±0.423**	**59**	**0.51±0.352**	**20**	**2.72**	**39**	**0.01**	**1.45**	**0.22**
LogEL	1.14±0.354	59	1.11±0.355	20	0.30	33	0.76	1.00	0.83
LogTL	2.30±0.424	59	2.16±0.442	20	1.21	32	0.23	1.08	0.81
LogFL	2.67±0.437	59	2.49±0.437	20	1.59	33	0.12	1.00	0.91
LogDNR	0.89±0.390	59	0.76±0.401	20	1.25	32	0.22	1.06	0.95
LogIMT	0.84±0.486	59	0.67±0.481	20	1.35	33	0.19	1.02	0.75

Values significantly different at the p<0.05 level are indicated in bold.

Significant differences were observed when comparing the linear equations of the regression lines for the variables SVL and DNR (Table 2), but not for SVL and END. This analysis reinforced the observed size variability between the morphotypes. As such, for every increase of one (1) millimeter of snout-vent length (logSVL) in the common morphotype, there was an increase of approximately 0.66 mm in the distance from the nostril to the most anterior extremity of the rostra (DNR). This relationship was even greater in the yellow morphotype, with the same DNR increasing almost 0.90 mm.

**Table 2:** Comparison test of the regression lines of measurements of the common and yellow morphotypes of *P. cristiceps*.

Variables	bc	SSc	Ba	SSa	Sb1–b2	/t/
SVL×END	0.19	6.08	0.18	2.47	0.042	0.24
**SVL×DNR**	**0.66**	**6.35**	**0.88**	**2.38**	**0.030**	**7.33**
SVL×IMT	0.26	6.33	0.22	10.04	0.050	0.80
SVL×FL	0.59	29.28	0.75	14.49	0.095	1.68
SVL×EL	0.21	8.91	0.30	5.01	0.054	1.67
SVL×TL	0.50	21.43	0.64	8.65	0.080	1.75
SVL×IND	0.15	5.18	0.16	2.70	0.040	0.25
SVL×HW	0.60	18.00	0.76	13.22	0.081	1.97

The variable emphasized corresponds to the one with a significant difference between the inclinations of the regression lines of the morphotypes, in critical values of the *t*_α(2),DF_ distribution: *t*_0,05(2),75_ = 1.92. SSc: sum of the squares of the common morphotype, Ssa: sum of the squares of the yellow morphotype, Sb1–b2: standard error, bc: regression coefficient of the common specimens, and ba: regression coefficient of the yellow specimens.

**Figure 1:**
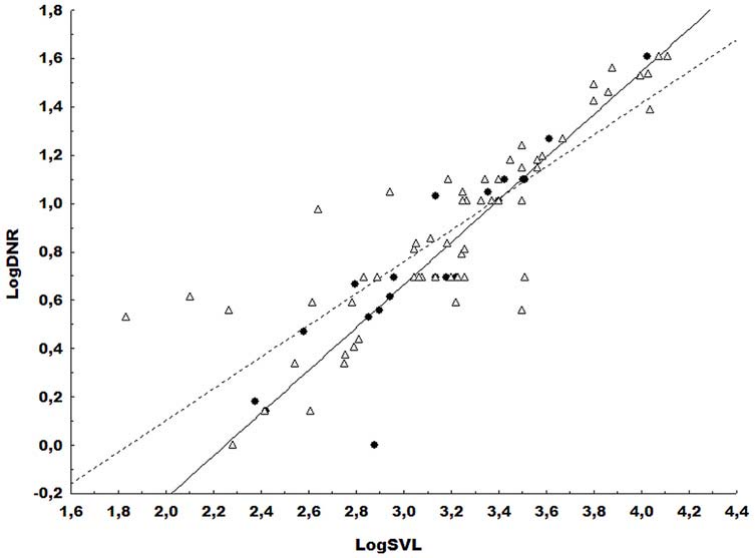
Graphical representation of the dispersal and regression lines. Variables: snout-vent length (SVL)×distance from the snout to the most anterior extremity of the rostra (DNR). The filled circles represent the yellow morphotype, and open triangles represent the common morphotype. Morphotype yellow Y = −1.98+0.88X, r = 0.91, p = 0.00000002 and Morphotype common Y = −1.21+0.66X, r = 0.85, p = 0.00000000. Std. error of estimate = 0.25; F(1.77) = 213.75.

The dispersion diagrams and the regression lines between snout-vent length (SVL) development in relation to the distance from the nostril to the most anterior extremity of the rostra (DNR) are presented in Figure 1.

It was observed that all of the variables had strong linear associations, and that the difference seen earlier in the *t* test in relation to the logarithmic variable eye-nostril distance (END) was reinforced by allometric studies of the morphotypes (Table 3). Discrete levels of variation were seen and the animals demonstrated similar patterns in terms of their overall body development (with the exception of the END variable).

**Table 3:** Allometric coefficients (±standard error) of the morphotypes of *P. cristiceps*.

Variables	Common morphotype (n = 59)			Yellow morphotype (n = 20)			t	DF	p	Variances	p Levene
	α	b	r	α	b	r					
HW	0.93±0.06	−0.23±0.08	0.86	1.21±0.16	−0.58±0.21	0.81	−0.01	31	0.99	1.17	0.62
IND	0.82±0.07	−0.86±0.09	0.78	0.78±0,12	−0.83±0.15	0.77	1.37	41	0.18	1.60	0.13
**END**	**0.84±0.07**	**−0.83±0.09**	**0.77**	**0.85±0.08**	**−0.92±0.10**	**0.92**	**2.72**	**39**	**0.01**	**1.45**	**0.22**
EL	0.70±0.05	−0.50±0.06	0.87	0.85±0.08	−0.70±0.10	0.92	0.30	33	0.76	1.00	0.83
TL	0.84±0.06	−0.17±0.05	0.85	1.50±0.21	−1.10±0.30	0.80	1.45	26	0.16	2.00	0.20
FL	1.03±0.08	−0.30±0.12	0.80	1.05±0.08	−0.34±0.11	0.94	1.24	39	0.22	1.43	0.77
DNR	0.80±0.05	−0.70±0.07	0.85	0.96±0.09	−0.97±0.12	0.91	1.25	32	0.22	1.06	0.95
IMT	0.96±0.06	−0.97±0.08	0.88	1.16±0.07	−1.3±0.09	0.96	1.35	33	0.19	1.02	0.75

Values significantly different at the p<0.05 level are indicated in bold.

**Figure 2:**
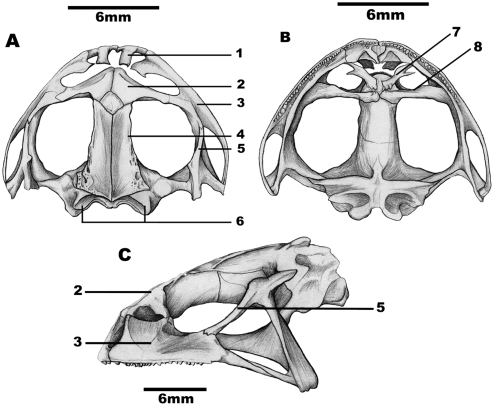
The cranium of a common morphotype specimen of *P. cristiceps*. A-dorsal view, B-palatal view, C-lateral view. 1-alary process, 2-nasal, 3-maxilla, 4-frontoparietal, 5-orbital branch of squamosal, 6-occipital condyle, 7-vomer and 8-palatine. Author: Kleber Vieira.

In comparing the crania, it was observed that in the common morphotype (Figure 2) the alary processes were long and narrow and the nasals were amply juxtaposed with a small contact area with the frontoparietals (these latter structures having small and un-accentuated crests and grooves situated in their posterior portion). The orbital branches of the squamosal were thick and long, and were sutured in their contact with the maxilla. The condyle occipitals were wide and not close, while the palatines were thin and made almost no contact with each other or with the vomers.

**Figure 3:**
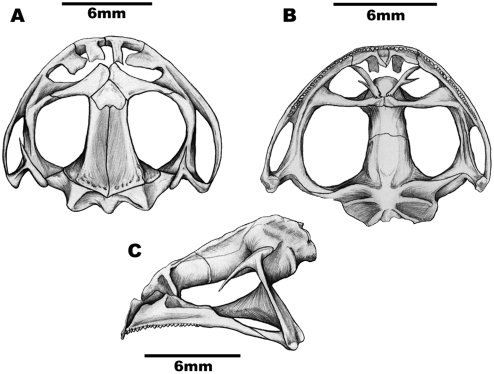
The cranium of a yellow morphotype specimen of *P. cristiceps*. A–dorsal view, B–palatal view, C–lateral view. Author: Kleber Vieira.

A relatively short cranium was observed in the yellow morphotype (Figure 3), with wide alary processes, as well as nasals juxtaposed at their anterior end. The condyle occipitals were wide and not close, the palatines touched each other as well as the vomers-to the contrary of the cranium of the common morphotype. The frontoparietals were short and moderately wide with no crests or grooves in their posterior portion, or when present, in very small numbers. The maxilla was concave and not sutured to the orbital branch of the squamosal in 67% of the individuals, and this latter structure was thin and cartilaginous near the maxilla. The characteristics observed were also present in specimens from other populations.

**Figure 4:**
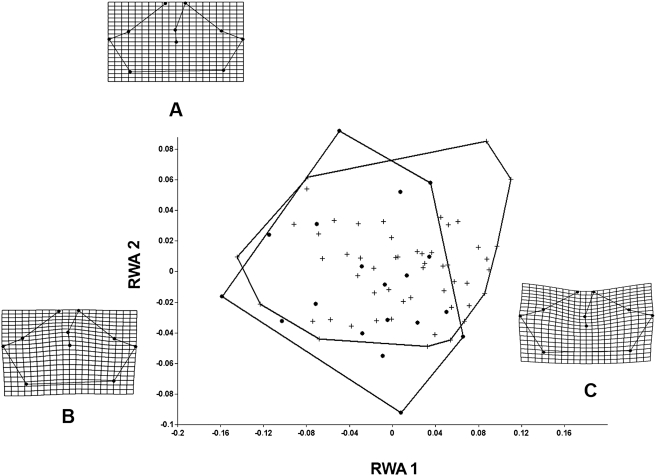
Relative Warp analysis of the morphotypes of *P. cristiceps* in dorsal configuration. Variance observed for the first two Relative Warps: Warp 1 (41.33%) and Warp 2 (26.50%). Circles: yellow morphotype; crosses: common morphotype. A–Mean Shape; B–Individual of *P. cristiceps* with a positive RWA 2 score and C–Individual of *P. cristiceps* with a positive RWA 1 score. Highly significant differences in Hotelling's t^2^ = 99.7; F(20.53) = 3.7; p: 0.000126.

**Figure 5:**
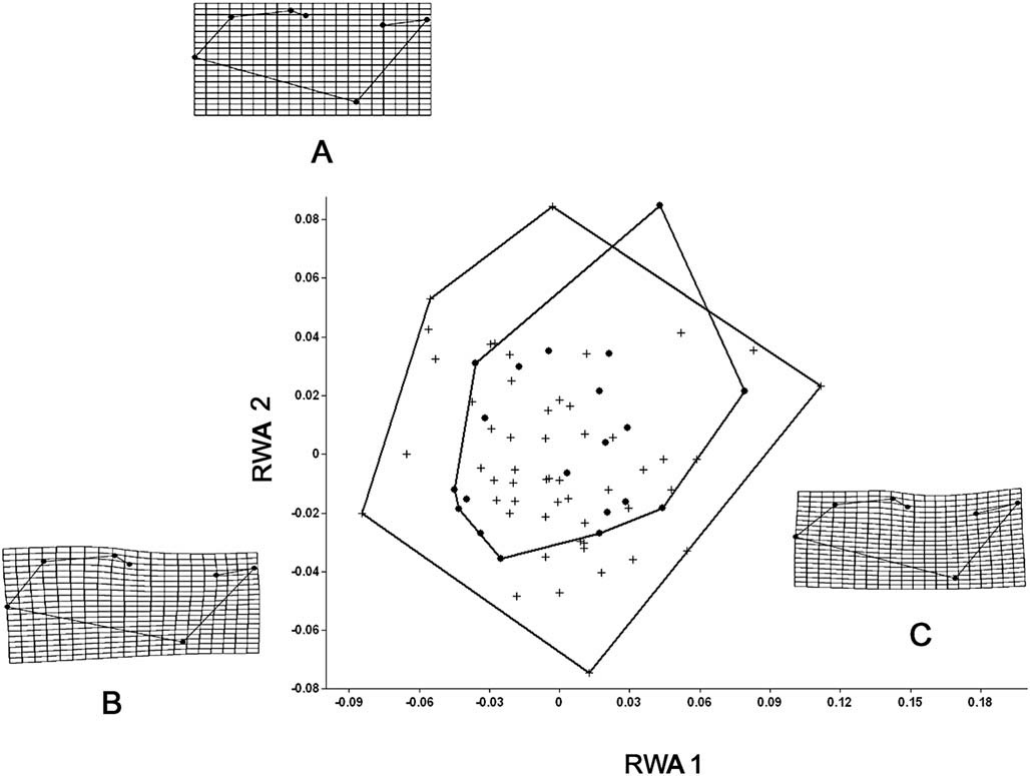
Relative Warp analysis of the morphotypes of *P. cristiceps* in lateral configuration. Variance observed for the first two Relative Warps: Warp 1 (27.92%) and Warp 2 (19.08%). Circles: yellow morphotype; crosses: common morphotype. A–Mean Shape; B–Individual of *P. cristiceps* with a positive RWA 2 score and C–Individual of *P. cristiceps* with a positive RWA 1 score. No significant differences were observed in Hotelling's t^2^ = 17.2; F(14.58) = 1.0; p: 0.71.

Figures 4 and 5 represent the RWA plots of the data sets of the dorsal and lateral configurations of the common and yellow morphotypes. In analyzing the two configurations it could be seen that the morphotypes were intimately related-which would be expected in studies of intrapopulational variation.

The differences in the dorsal configurations were principally related to landmarks 1, 2, 5, 8, and 9 in individuals of the common morphotype, and were characterized by a shift to the right in the RWA 1 (the opposite of that seen in the yellow morphotype). In the yellow morphotype, there was a shift to the left, with variations of landmarks 3, 5, 6, and 7. The principal differences between the common and yellow morphotypes can be seen in the occipital, supra-ocular, and rostral regions. The Hotelling's *t*-test, of discriminate function, suggests that these differences are significant.

The analyses of the lateral configurations of the specimens were more uniformly distributed, and in spite of the fact that certain differences were observed they were not statistically significant by the Hotelling's *t*-test of discriminate function.

## Discussion

Two basic types of variation can be considered from an evolutionary point of view [9]: group variation, which refers to differences between populations; and individual variation, which considers the differences between individuals of a single population (including polymorphic variations in which any physical trait can vary individually, whether it be an external morphological character, a physiological attribute, or even related to structural cytology-such as the number, pattern, and/or form of the chromosomes). This variation can result in the simultaneous occurrence of various genetic factors (alleles or genetic dispositions) in a population-resulting in discontinuous phenotypic effects, which in many cases would be very common in the homologous polymorphic series typical of certain animal families.

In considering metric characteristics (such as external morphological attributes), a given animal population can demonstrate relative degrees of variability, especially when their shape is considered a valid character for analysis. This can be seen in the specimens of *P. cristiceps* examined here, which can readily be classified into two color categories-a typical example of the intraspecific and intrapopulational variability. Additionally, as was demonstrated above, this same variation can be quantified using metric and shape attributes.

The animals of the yellow morphotype category generally have smaller dimensions that the common morphotype, and as demonstrated by the Student *t* test one of the measurements was actually statistically significant, showing a certain degree of cephalic variation.

Reinforcing the observed differences between the color morphotypes, the cranial characters analyzed demonstrated that the yellow morphotype has a relatively short skull, which is supported by the statistically significant differences observed in the *t* test, in the eye-nostril distance (END), as well as in the linear equation of the distance from the nares to the most anterior extremity of the rostra (DNR). The morphotypes behave like differentiated elements in terms of the DNR criterion, as would be expected for representatives of disjoint populations [10]. Additionally, the frontoparietals and the alary process were short and wide in the yellow morphotype, with the orbital branch not being sutured to the maxilla in most of the individuals analyzed-a characteristic that is considered generic to *Odontophrynus* Reinhardt & Lütken, 1862 [4]. The common morphotype, to the contrary, demonstrates a more elongated cranium and the squamosal orbital branch is thick and sutured to the maxilla-this latter characteristic being typical of the genus *Proceratophrys* [11].

Variations in bone structure among individuals of the same species are quite common, principally when considering the absence or presence of certain cranial structures. Intraspecific variability in the number and morphology of the lacrimal, sclerotic ossicles, and maxilla has been described in some species of lizards of Iberian Peninsula [12], while in another study with marsupials, cranial variability was observed principally in the nasals, postorbital processes, and supra-orbital crests [13]. Generally these types of variations can originate with the process of ossification during development, as noted in amphibians of the species *Ascaphus truei* [14]; this type of variability is uncommon in mammals, however, as skeletal characteristics tend to be conservative.

The absence of bony structures or even differences in the number of bones, principally when accompanied by chromatic variability, can generate problems for the systematics of some taxa-and in order to avoid taxonomic errors it is necessary to recognize certain characters as being valid and take into account the magnitude of those variations. In examining cranial variability in *Erinaceus concolor* [15], the types and magnitudes of the differences in the nasomaxillary suture and cranial morphology pointed to the existence of morphotypes and not different species, as has often been observed in other groups.

The morphotypes of *P. cristiceps* are distinguished not only by a number of cranial characteristics but also by studies of cranial shape. Our analyses demonstrated that the principal variations were specifically located in the occipital, supra-ocular, and rostral regions, as well as in the prootic-squamosal length, giving rise to the two distinct morpho-shapes. The use of geometric morphometry identified valid differences between the animals and a more precise understanding of the magnitude of variation between the common and yellow phenotypes, completing the univariate analysis of the data. The utilization of this type of analysis is critical to understanding the interactions between phenotype, genotype, and environmental spaces [16], principally in studies of amphibians.

The identification of polymorphism in individuals of any species requires care in choosing the focal characters, as well as in defining the parameters of that polymorphism. Polymorphism is a variation within a species that is independent of ontogeny and of the sex of the animal (assuming that the variation is genetically based and hereditable) [17]. Adopting this definition, characteristics such as eye-nostril distance, distance from the nares to the most anterior extremity of the rostra, aspects of head shape, as well as variations in cranial bones, do in fact correspond to polymorphic characters because of the existence of significant statistical differences between the morphotypes.

Even though they demonstrated a certain degree of variability, many characteristics beyond those related to external morphology were observed to be held in common among the morphotypes of *P. cristiceps*. A good example is the similarity in the pattern of overall body development evident in the allometry analysis-which represents a quantitative phenomenon typical of the larger population, and whose distortions were only perceived when the animals were submitted to a much more profound analysis (such as the slope of the regression lines or multivariate shape analyses) that supported the differences between the morphotypes.

Individuals of the species *P. cristiceps* were seen to have a series of variations beyond those normally recognized (such as general color pattern), and we therefore considered the morphological divergence of the coloration patterns in populations as being due to environmental or genetic fluctuations over time [18]. These differences are clear and reinforce the existence of at least two morphotypes within that species. This phenomenon is not exclusive to the population found at the Pedra da Boca State Park-as the yellow morphotype is also known from other localities–and could be verified by cranial analyses of individuals from other populations and from herpetological collections. As such, the variations seen in the specimens examined do not represent local or isolated events.

## Materials and Methods

**Figure 6:**
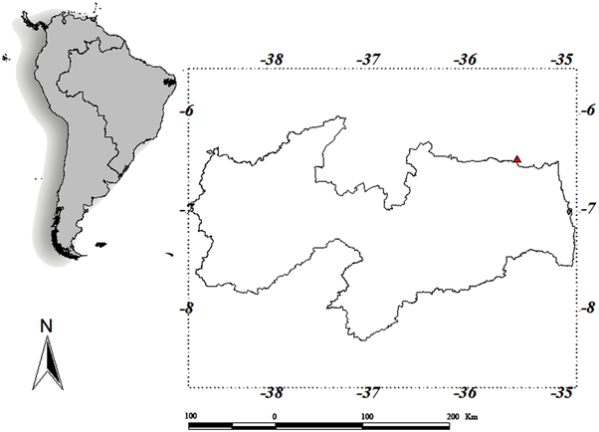
Location of the Pedra da Boca State Park (red triangle).

A total of 79 specimens of *P. cristiceps* were collected using both active and passive capture techniques in the Pedra da Boca State Park (Figure 6) (6°46′18″S×35°38′80″W). The Park is located in the municipality of Araruna, in the Vale do Curimataú Oriental geographic meso-region of the Borborema, in northern Paraíba State, Brazil.

Active collections were undertaken in November 2003 and between January and April 2004. Passive collections were made near small streams using interception and pitfall traps [19]. The captured specimens were euthanized following international norms [20] and then fixed in 4% formalin, and subsequently preserved in 70° GL alcohol. All specimens were deposited in the Herpetological Collection of the Department of Systematics and Ecology, Centro de Ciências Exatas e da Natureza, Universidade Federal da Paraíba, Brazil.

**Figure 7:**
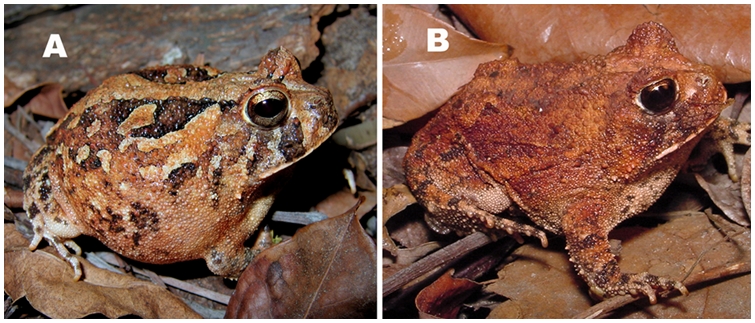
Morphotypes of *P. cristiceps* encountered in the Pedra da Boca State Park. A–Common morphotype; B–Yellow morphotype. Photographs: Cláudio Sampaio (A) and Yuri Lima (B).

Of the 79 specimens of *P. cristiceps* collected, 59 (75%) could be characterized as the “common” morphotype while the other 20 (25%) were of the “yellow” morphotype (Figure 7). The “common” morphotype has the typical coloration attributed to the species-from brown to gray, while the “yellow” (or an approximate tone) morphotype is less frequently encountered. The term morphotype, as used here, follows the definition for certain characteristics within a population that are derived from genetic or ontogenetic factors [21].

Specimens of both morphotypes were compared with the original description of the species *P. cristiceps*, as well as with additional characteristics presented by subsequent authors [4], [11], [22], in order to confirm the identities of the study material [23]. Additionally, the animals collected in the State Park were compared with specimens securely identified by M. T. Rodrigues and P. Cascon as being *P. cristiceps*, and which had previously been deposited in the Herpetological Collection of the Systematics and Ecology Department, CCEN/UFPB.

Twenty-four crania were prepared and examined-twelve from the common morphotype and twelve from the yellow variety. All of the skulls were from adult individuals (SVL greater than 30 mm) according to established classification [24], [25]. The animals were dissected and the cephalic portion was removed, and the crania were subsequently cleaned with a 3% KHO solution [26]. After cleaning and removal of the jaws, the skulls were placed in a drying oven for two days. Drawings were made of the dorsal, palatal, and lateral views of the dry material using a *camara lucida* apparatus coupled to a stereomicroscope. All of the bone reference points, as well as their nomenclature, likewise followed established classifications [27], [28].

Fourteen crania of *P. cristiceps* (seven from the common morphotype and seven from the yellow variety) from Palmeirais (5°58′40″S×43°03′48″) in the Parnaíba River basin, Piauí State, and from areas within the municipalities of Patos (7°01′28″S×37°16′48″W) and Cabaceiras (7°22′50″S×36°17′51″W) in Paraíba State, were examined and compared with specimens collected in the Pedra da Boca State Park. Of the seven animals of the common morphotype, two were from Patos, two from Cabaceiras, and three from Palmeirais. Of the seven animals of the yellow morphotype, three were from Palmeirais, three from Cabaceiras, and one from Patos.

### Morphometric studies

Precision (0.05 mm) Veneer calipers were used to measure all of the 79 specimens collected. Nine different measurements were made: snout-vent length (SVL), head width (HW), internarial distance (IND), eye-nostril distance (END), eye length (EL), tibia length (TL), foot length (FL), length of internal metatarsal tubercle (IMT), as well as the distance from the snout to the most anterior extremity of the rostra (DNR). These measurements were chosen as being the most informative and the most frequently used by taxonomists working with this group.

All measurements were subjected *a priori* to the Shapiro-Wilks normality test (data not shown) using the Statistica software package (StatSoft; Inc.; OK) in order to confirm their normal distribution. The Shapiro-Wilks test was used due to its adaptability to a wide range of problems related to evaluating the normality of univariate data [29]. The variables were subsequently log-transformed (natural log) and the Student *t* test applied to compare the measurement averages between morphotypes. When the absolute value between the two measurements was equal or greater than the minimal significant difference (MSD), those measurements were considered statistically different at a p<0.05 significance level [30]. The Levene's test was used to determine if the variables were homoscedastic or not.

Regression analysis was also applied to the SVL (and other measurements) in order to compare the linear equations of the independent (SVL) and dependent variables through comparison tests of their regression lines [10], [31]. We also estimated the allometric coefficients using the reduced major axis (RMA) and calculated their standard error [32]. Using the residual regression values that were calculated by the regression analysis used in obtaining the allometric coefficients, we performed the Student *t* test to determine if the differences between the morphotypes were statistically significant.

The analyses of specimen shapes were undertaken considering the cephalic area in dorsal and left-lateral views. Head shape was chosen as this character is considered a determinant factor in studies of intra- and interspecific variation in the genus *Proceratophrys* [24].

The choice of the landmarks used with the specimens of *P. cristiceps* from the Pedra da Boca State Park was based on diagnostic characters traditionally used for the different species of this genus [25], [33]–[36]. In addition to representing important characteristics in studies of these taxa, all of the points chosen represent phylogenetic and ontogenetic homologs among the specimens, according to the criteria of topographic equivalence in primary homology [37]. This method was applied to diminish errors among correspondence points [38]–[41]. As such, we analyzed 73 individuals of both morphotypic varieties, including 53 individuals identified as the common morphotype, and 19 individuals of the yellow morphotype. Of this total, six specimens of the common morphotype and one specimen of the yellow morphotype were later excluded due to evidence of injuries to these animals.

**Figure 8:**
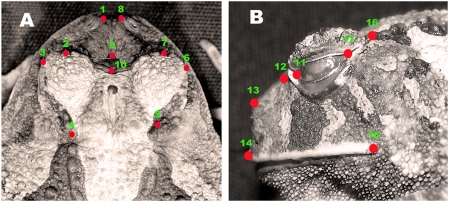
Landmarks locations on *P. cristiceps*. A-dorsal view and B-lateral view. Photographs: Washington Vieira.

Ten anatomical landmarks in the dorsal midline plane (Figure 8A) were selected in all specimens: 1–the most anterior extremity of the left nasal crest; 2–the extreme-left nodule of the anterior amphiocular crest; 3–the most prominent left supra-ocular nodule; 4–the first nodule of the left oculodorsal crest (situated immediately posterior to the left orbit); 5–the first nodule of the right oculodorsal crest (situated immediately posterior to the right orbit); 6–the most prominent right supra-ocular nodule; 7–the extreme-right nodule of the anterior amphiocular crest; 8–the most anterior extremity of the right nasal crest; 9–the extreme-anterior nodule of the anterior amphiocular crest (equivalent in its position to the median-central portion of the sphenethmoid); and 10–the median tubercle of the anterior amphiocular crest (equivalent in its position to the median-central portion of the frontoparietals that make contact with the sphenethmoid).

For the lateral cephalic region (Figure 8B) the landmarks used were: 11–the extreme-anterior margin of the eye; 12–the extreme-posterior of the canthal crest (tubercles at the anterior border of the eyelid); 13–the most anterior extremity of the nasal crest; 14–the most anterior extremity of the upper lip (equivalent in position to the extreme-anterior portion of the pars dentalis); 15–extreme-posterior of the upper lip; 16–the first nodule of the oculodorsal series; and 17–the extreme-posterior margin of the eye. The coordinates of the landmarks of each specimen were obtained using the tpsDig software package [42].

We used the method of point super-positioning following optimization criteria (Procrustes) during shape analyses to generate the smallest possible sum of the squares of the distances between corresponding points [43]. This method is considered the most reliable for determining morphometric relationships in shape analysis [44]–[46].

In order to determine the relationships between the shapes of the two morphotypes of *P. cristiceps* we calculated a RWA with α = 0 and assigned equal importance to all of the spatial scales (as this is the best option for exploratory studies) [47]. We then used the Thin-plate spline methodology to examine the variation between the morph-forms. The relative deformation method functioned as an optimal alternative for studies of intrapopulational variation [48].

In order to determine if both of the distance shapes of the morphotypes were statistically significant, a discriminate function analysis was performed with the values transformed into Procrustes residuals by subtracting the mean shape, and a *P*-value was calculated using Hotelling's *t*-test function at a significance level of p<0.001. This latter method was applied as it represents a natural multidimensional extension of the *t*-statistic widely used in the biological sciences [49]–[52]. The Procrustes superimposition and Thin-plate spline method were performed using the Past software package [53], while the Hotelling's *t*-test and discriminate functions were generated using Statistica software (StatSoft; Inc.; OK).
